# E2F8, a direct target of miR-144, promotes papillary thyroid cancer progression via regulating cell cycle

**DOI:** 10.1186/s13046-017-0504-6

**Published:** 2017-03-07

**Authors:** Jing Sun, Run Shi, Sha Zhao, Xiaona Li, Shan Lu, Hemei Bu, Xianghua Ma, Chuan Su

**Affiliations:** 1 Department of Endocrinology, The First Affiliated Hospital of Nanjing Medical University, 300 Guangzhou Road, Nanjing, 210029 China; 2 The Fourth Clinical College of Nanjing Medical University, Hanzhong Road 140, Nanjing, 210029 China; 3 Department of Pathology, The First Affiliated Hospital of Nanjing Medical University, 300 Guangzhou Road, Nanjing, 210029 China; 4 Health Management Center, The First Affiliated Hospital of Nanjing Medical University, 300 Guangzhou Road, Nanjing, 210029 China; 5 Department of Nutriology, The First Affiliated Hospital of Nanjing Medical University, 300 Guangzhou Road, Nanjing, 210029 China; 6 Department of Pathogen Biology and Immunology, Jiangsu Key Laboratory of Pathogen Biology, Nanjing Medical University, 101 Longmian Avenue, Jiangning District, Nanjing, 211166 China

**Keywords:** E2F8, miR-144, Papillary thyroid cancer (PTC), TCGA, Proliferation, Cell cycle

## Abstract

**Background:**

Thyroid cancer is the most common malignancy of endocrine system, and papillary thyroid cancer (PTC) is the most common subtype. E2F8, a novel identified E2F family member, was reported to associate with progression of several human cancers, however, its clinical significance and biological role in PTC remain unknown.

**Methods:**

E2F8 or miR-144 expression profiles in PTC tissues were obtained from The Cancer Genome Atlas (TCGA) datasets, and the correlation of E2F8 expression with clinicopathological features was analyzed in a cohort PTC patients. The effects of E2F8 and miR-144 on proliferation were evaluated both in vitro and in vivo. Luciferase reporter assay was used to determine E2F8 was a direct target of miR-144.

**Results:**

E2F8 was widely upregulated in PTC tissues, and overexpression of E2F8 was correlated with more aggressive clinicopathological features. In contrast, we found that silence of E2F8 significantly suppressed proliferation of PTC cells by inducing G1-phase arrest via downregulating Cyclin D1 (CCND1) both in vitro and in vivo. We also identified miR-144 as a tumor-suppressive microRNA that directly targeted E2F8 to inhibit proliferation of PTC cells in vitro and in vivo. Moreover, miR-144 was widely downregulated in PTC, where its expression correlated inversely with E2F8 expression.

**Conclusions:**

Our results demonstrate a new miR-144/E2F8/CCND1 regulatory axis controlling PTC development, which may offer a potential prognostic and therapeutic strategy.

**Trial registration:**

No applicable.

**Electronic supplementary material:**

The online version of this article (doi:10.1186/s13046-017-0504-6) contains supplementary material, which is available to authorized users.

## Background

Thyroid cancer is the most common malignancy of endocrine system, and its incidence has increased rapidly worldwide in the past few decades [1]. Thyroid cancer is classified into four types: papillary, follicular, medullary and anaplastic thyroid cancer. Papillary thyroid cancer (PTC) is the most common subtype, accounting for more than 80% of all thyroid cancers, and associates with a favorable therapeutic response and prognosis [2]. However, in the case of aggressive PTC and certain PTC variants, a regional recurrence or distant metastasis is observed in 5–20% of the patients who have undergone total thyroidectomy [3, 4]. Therefore, novel biomarkers and potential therapeutic targets are eagerly needed to provide better follow-up treatment.

As known, E2F family function as transcription factors that bind to target promoters and regulate their expressions [5]. To date, eight members, E2F1-8, have been recognized. In general, E2F1-3 are considered as transcriptional activators, whereas E2F4-7 play an inhibitory role in transcriptional expression of downstream target genes [6–8]. However, the function of a novel member, E2F8, is still poorly understood. It has been reported that E2F8, in combination with E2F7, is required for embryonic development in mice [9, 10], angiogenesis [11] and lymphangiogenesis [12] in zebrafish. Moreover, E2F8 expression has been found to be upregulated in ovarian cancer [13], hepatocellular cancer [14], lung cancer [15] and breast cancer [16]. Furthermore, E2F8 also promoted cancer malignant progression in breast cancer [16], prostate cancer [17] and hepatocellular cancer [14], and served as a therapeutic target in lung cancer [15]. These findings have indicated that E2F8 might play a role in the development of cancer. However, the clinical significance and biological function of E2F8 in PTC has not yet been investigated.

MicroRNAs (miRNA) are small noncoding RNAs that can modulate the expression of cognate target genes by binding to their mRNA 3’-untranslated region (3’-UTR), resulting in either translational inhibition or mRNA cleavage [18]. Accumulating evidence indicates that miRNAs can act in human carcinogenesis as novel types of tumor suppressors or oncogenes [19–21]. Several studies have shown that miRNAs have an important role in PTC progression [22–24]. Hence, we are interested in whether E2F8 expression is regulated by certain miRNAs as a posttranscriptional regulation mechanism in PTC.

In this study, we provide the first evidence that E2F8 overexpression is associated with more aggressive clinicopathological features in PTC. We show that silence of E2F8 inhibited proliferation of PTC cells both in vitro and in vivo. Moreover, we demonstrate that E2F8 can promote G1/S transition via upregulating Cyclin D1 (CCND1) at least. We further demonstrate that E2F8 is a direct functional target of miR-144, which controls PTC cell proliferation both in vitro and in vivo. Our results suggest that the miR-144/E2F8/CCND1 axis might function as a key pathway regulating tumor cell proliferation during PTC development.

## Methods

### Data sources and bioinformatics

Two TCGA datasets named *TCGA_THCA_miRNA_HiSeq-2015-02-24* and *TCGA_THCA_exp_HiSeqV2-2015-02-24* were downloaded at the website of the UCSC cancer browser (https://genome-cancer.ucsc.edu/) [25], containing 59 paired PTC tissues and adjacent normal tissues. All normalized gene expression values can be obtained from “genomicMatrix” files.

A list of 143 genes with highest co-expression correlation (Pearson *r* value > 0.5) (Additional file 1: Table S1) with E2F8 were submitted to DAVID Bioinformatics Resources 6.7 (http://david.abcc.ncifcrf.gov/) [26] for Gene Ontology (GO) enrichment analysis.

### Tissue collection

In this study, we collected 64 paired cases of PTC and adjacent normal tissue samples from patients who underwent surgical resection at The First Affiliated Hospital of Nanjing Medical University (Nanjing, China) from 2012 to 2015. Informed written consent for scientific use of biological material was obtained from each patient, and this study was approved by the Ethics Committee of Cancer Institute of Jiangsu Province. All patients’ clinicopathological parameters, including age, gender, primary tumor size, lymph node status, TNM stage, tumor location and focus type, were obtained from their medical records.

### Cell culture and transfections

BCPAP and TPC-1 cells were cultured in RPMI1640 media (KeyGEN, Nanjing, China) supplemented with 10% fetal bovine serum and penicillin/streptomycin, and cultured at 37 °C in a humidified incubator containing 5% CO2. Transfection was performed following the small-interfering RNA (siRNA) sequences transfection protocol for Lipofectamine RNAi MAX (Invitrogen, USA). Nonsense RNAi (nsRNA) was used as a negative control. Transfection efficiency was evaluated by quantitative real-time RT-PCR and western blot. miR-144 mimic, control mimic, control inhibitor, miR-144 inhibitor and siRNAs against E2F8 were synthesized by Genechem. The sequences used were: siRNA-1 for E2F8: 5’-GGCCAAAGACUGUAUACACTT-3’(sense), 5’-GUGUAUACAGUCUUUGGCCTT-3’(antisense); siRNA-2 for E2F8: 5’-GCCCUAUCAAGACCAACAATT-3’(sense), 5’-UUGUUGGUCUUGAUAGGGCTT-3’(antisense). And the following nonsense siRNA was used as negative control (NC): 5’-UUCUCCGAACGUGUCACGUTT-3’(sense), 5’-ACGUGACACGUUCGGAGAATT-3’(antisense). miR-144 mimic: 5’-UACAGUAUAGAUGAUGUACU-3’. The human E2F8-targeting small hairpin RNA sequences were designed based on siRNA-1 and nsRNA. We generated recombinant lentiviral particles and cells were transfected with E2F8 or negative control recombinant lentivirus (shRNA-E2F8 or shRNA-NC, respectively). For overexpressing miR-144, recombinant lentiviruses containing miR-144 precursor or negative control sequences were purchased from Genechem. For overexpressing CCND1 and E2F8, CCND1 cDNA and E2F8 cDNA without 3’-UTR were cloned into a pEGFP-N1 vector (purchased from Genechem) to construct overexpression plasmid, and an empty vector (EV) was used as a negative control.

### Luciferase reporter assay

A wild-type 3’-UTR fragment of E2F8 cDNA was amplified by using PCR and cloned into XbaI and SacI site of pmirGLO dual-luciferase miRNA target expression vector (Promega, Madison, WI, USA) and named as WT-E2F8 3’-UTR. The mutant variant of E2F8 3’-UTR was generated based on WT-E2F8 3’-UTR by mutating six nucleotides that potentially bind to miR-144 and named as Mut-E2F8 3’-UTR. These vectors (WT-E2F8 3’-UTR or Mut-E2F8 3’-UTR were together with miR-144 mimic or miR-NC) were transiently transfected into BCPAP and TPC-1 cells using Lipofectamine 2000 reagent (Invitrogen). Luciferase activity was measured using the Dual-Luciferase Reporter Assay System (Promega, Madison, USA) after transfection at 48 h. Data are presented as the mean value ± SD for triplicate experiments.

### RNA extraction and quantitative real-time(qRT)-PCR

Total RNA was extracted from cultured cells using TRIzol reagent (Invitrogen, Carlsbad, CA, USA) according to the manufacturer’s instruction. The qRT-PCR data collection was performed using a QuantStudioTM 6 Flex Real-Time PCR System and the qRT-PCR reaction included an initial denaturation step at 95 °C for 10 min, followed by 40 cycles of 92 °C for 15 s and 60 °C for 1 min. The primers are shown in Additional file 2: Table S2. Each sample was run in triplicate and the relative expression was calculated and normalized using the 2^-ΔΔCt^ method.

### Protein preparation and western blot

Cells were harvested and treated with lysis buffer on ice (KeyGEN, Nanjing, China), and a BCA kit (KeyGEN, Nanjing, China) was used to quantify protein concentration. Equal amounts of protein were loaded in SDS–PAGE gels. After separation in the gel, the protein was transferred on a PVDF membrane. Membranes were blocked in 2% BSA in TBS-T for 1 h, and then incubated overnight (4 °C) with antibodies against E2F8 (Abcam, ab185727 1:1000), cyclin D1 (CST, 2978 1:1000) or β-actin (Cell Signaling, 8H10D10 1:1000). After being washed in TBS-T, membranes were incubated with goat anti-rabbit HRP-conjugated secondary antibody (1:10,000; Abcam) or goat anti-mouse HRP-conjugated secondary antibody (1:10,000; Abcam) for 2 h at room temperature. The blots were visualized by ECL detection (Thermo Scientific). All experiments were repeated at least three times independently.

### TMA and immunohistochemistry

A tissue microarray (TMA) containing 58 paired formalin-fixed paraffin-embedded (FFPE) PTC and adjacent normal tissue samples was used. The TMA was purchased from the Shanghai Biochip Co., Ltd., Shanghai, China. All tissues were re-examined by an experienced pathologist after they were transferred from a local hospital and the TNM stage was determined in each patient.

Immunohistochemistry(IHC) for E2F8 protein expression in samples was performed using standard methods. Briefly, tissue sections were deparaffinized and rehydrated through graded alcohol. Endogenous peroxidase activity was blocked by incubation in 3% H_2_O_2_. Antigen retrieval was carried out with 0.01 M citrate buffer (pH 6.0) and microwave heat induction. E2F8 staining was scored by blinded observers (including a pathologist) according to intensity and percentage of positive cells. The staining intensity was scored according to 4 grades: 0 (No staining), 1 (weak staining), 2 (intermediate staining), or 3 (strong staining). The product (percentage of positive cells and respective intensity scores) was used as the final staining score (a minimum value of 0 and a maximum of 300).

### Cell proliferation assay

The cell proliferation was monitored using a Cell Counting Kit-8 (KeyGEN, Nanjing, China) or the xCELLigence system. After transfection, cells were plated in 96-well plates at a density of 2000 cells in 100ul per well and the absorbance was measured at 450 nm with an ELx-800 Universal Microplate Reader. Experiments were repeated at least three times with similar data. For the xCELLigence system, exponentially growing cells with corresponding treatment in complete media were seeded in E-plates at a density of 20,000 per well. The plates were then locked into the RTCA DP device in the incubator. The proliferative ability in each well was automatically monitored by the xCELLigence system and expressed as a “cell index” value. The cell growth was recorded in real-time for 90 h.

For colony formation assay, a total of 100 transfected cells were placed in a fresh 6-well plate and maintained in media containing 10% FBS, replacing medium every 3 or 4 days. After 2 weeks, cells were fixed with 4% paraformaldehyde and stained with 0.1% crystal violet. Visible colonies were then counted. For each treatment group, each well was assessed in triplicate.

### Cell cycle analysis

Flow cytometry analysis was performed to detect cell cycle distribution. Cells were transferred and fixed in centrifuge tubes containing 4.5 mL of 70% ethanol on ice. The cells were kept in ethanol for at least 2 h at 4 °C. Then, the ethanol-suspended cells were centrifuged for 5 min at 300 g. Cell pellets were resuspended in 5 mL of PBS for approximately 30 s and centrifuged at 300 g for 5 min, then resuspended in 1 mL of PI staining solution and kept in the dark at 37 °C for 10 min. Samples were analyzed using a FACSCalibur flow cytometer. The percentage of the cells in G0–G1, S, and G2–M phases were counted and compared. All the samples were assayed in triplicate.

### Xenograft experiment

All animal studies were conducted in accordance with NIH animal use guidelines and protocols approved by Nanjing Medical University Animal Care Committee. Twelve male nude mice (ages 4–6 weeks) were purchased from Nanjing Medical University School of Medicine’s accredited animal facility. Briefly, in each group, 1.0 × 10^6^ exponentially growing TPC-1 cells were injected in axilla subcutaneously. Tumor volume was estimated using calipers every week as length × width^2^ × 0.5. Five weeks after injection, mice were sacrificed, tumor weights were measured and tumors were collected for further analysis.

### Statistical analysis

All statistical analyses were performed using SPSS Statistics (version 20.0, Chicago, Ill) and GraphPad Prism 6 software (GraphPad Software, Inc., La Jolla, CA, USA). The results were presented as mean ± S.D. Relative quantification of mRNA expression level was calculated with the 2^-ΔΔCt^ method. Student’s *t*-test was used to analyze difference between two groups. Correlation of E2F8 expression with other genes was analyzed using Pearson test. Association of E2F8 expression with clinicopathological parameters was analyzed using Chi-square test. *p* < 0.05 was considered statistically significant.

## Results

### Upregulation of E2F8 correlates with more aggressive clinicopathological features in PTC

**Fig. 1:**
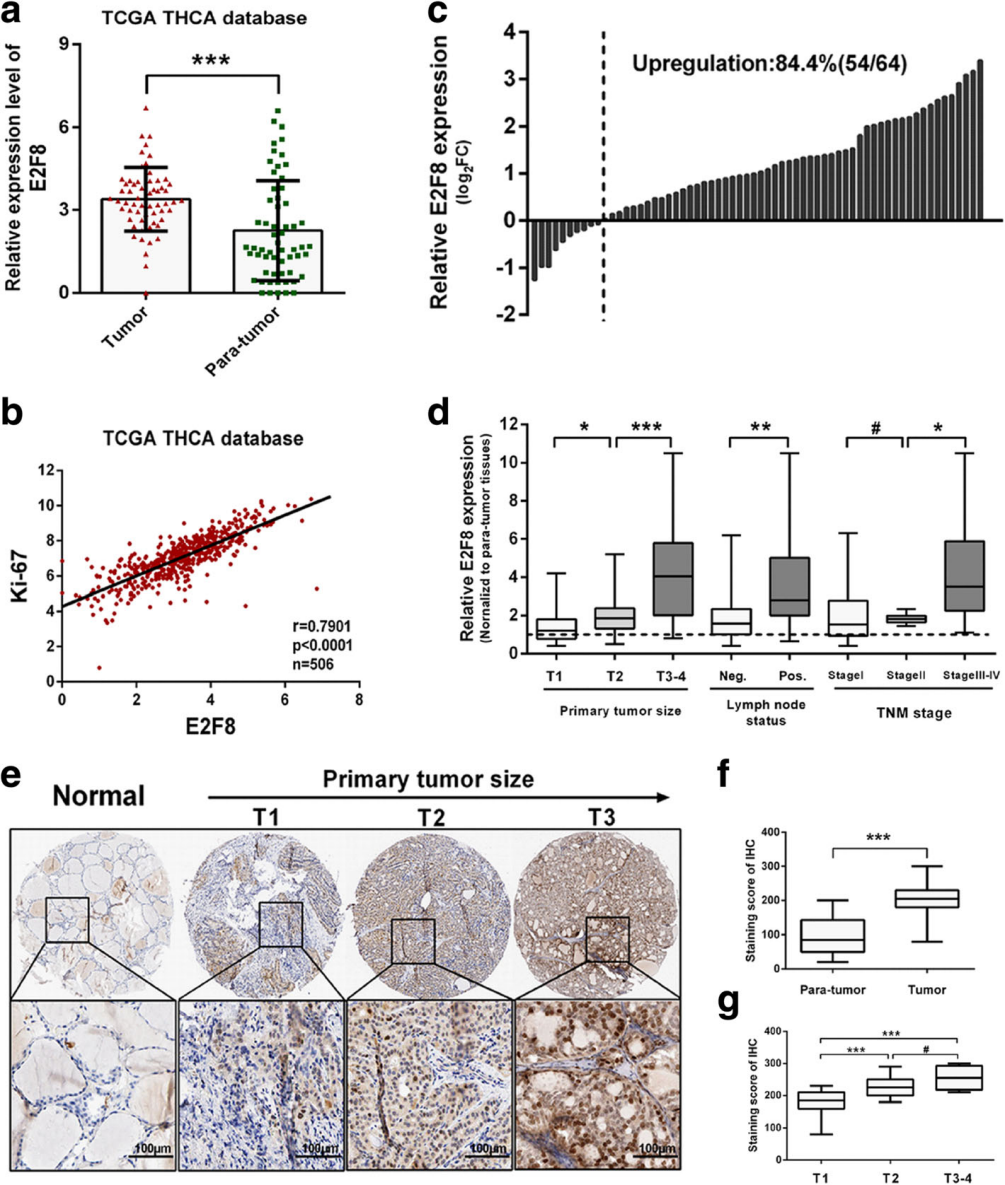
E2F8 is widely upregulated in PTC tissues and correlates with more aggressive clinicopathological features. **a** TCGA dataset showed that E2F8 was significantly upregulated in PTC tissues compared with adjacent normal tissues. **b** TCGA dataset showed E2F8 was positively correlated with Ki-67(*r* = 0.7901, *p* < 0.0001, *n* = 506) in PTC tissues. **c** qRT-PCR analysis showed that E2F8 was upregulated in 84.4% of 64 PTC patients (Normalized to adjacent normal tissues). **d** E2F8 mRNA fold change in different T stages, N stages and TNM stages. **e** Representative TMA IHC analysis of E2F8 in normal thyroid tissue and PTC tissues in different T stages. **f** E2F8 staining score was significantly increased in PTC tissues compared with adjacent normal tissues. **g** E2F8 staining score was significantly increased along with more advanced T stage in PTC tissues. *p < 0.05, **p < 0.01, ***p < 0.001, # No significance

Analysis of the *TCGA_THCA_exp_HiSeqV2-2015-02-24* dataset showed that the mean expression value of E2F8 was increased in PTC tissues compared to adjacent normal tissues (3.389 ± 0.1505 vs 2.257 ± 0.2352; *p* < 0.001) (Fig. 1a). Moreover, Pearson correlation test in TCGA PTC dataset showed that E2F8 was positively correlated with Ki-67(*r* = 0.7901, *p* < 0.0001, *n* = 506), a frequently used proliferation marker (Fig. 1b).

The expression profile of E2F8 was then further validated by qRT-PCR in 64 paired PTC patients’ tissues (tumor and adjacent normal tissues). As shown in Fig. 1c, E2F8 mRNA was upregulated in 84.4% (54 out of 64) of PTC patients. To associated E2F8 mRNA expression with clinicopathological features (including age, gender, primary tumor size, lymph node status, TNM stage, tumor location and focus type), patients were divided into two groups: low-E2F8 and high-E2F8 group, according to the median expression level. We found that high E2F8 level was significantly associated with bigger tumor size(*p* = 0.0025), positive lymph node status(*p* = 0.0244) and advanced TNM stage(*p* = 0.0166) (Table 1). As shown in Fig. 1d, Student’s *t*-test also exhibited similar results. However, no significant association was observed between E2F8 expression with age, gender, tumor location or focus type.

**Table 1:** Correlation between E2F8 expression and clinical characteristics(*n* = 64)

Characteristics	E2F8-low cases	E2F8-high cases	*P*-value
Age at diagnosis(years)			0.2093
≤ 45	20	15	
> 45	12	17	
Sex			0.2661
Male	11	7	
Female	21	25	
Location			0.4334
Left lobe	15	11	
Right lobe	13	19	
Bilateral	3	1	
Isthmus	1	1	
Focus type			0.0696
Unifocal	22	28	
Multifocal	10	4	
Tumor size			0.0025^a^
T1-T2	24	12	
T3-T4	8	20	
Lymph node status			0.0244^a^
N0	20	11	
N1	12	21	
Tumor stage			0.0166^a^
I-II	26	17	
III-IV	6	15	

^a^Significant correlation

To evaluate the protein expression of E2F8 in PTC tissues from a cohort of 58 papillary thyroid cancer patients, immunohistochemistry(IHC) was performed using specific anti-E2F8 antibody. As results, we observed that E2F8 was predominantly localized in the nuclei of PTC cells. Significantly higher expression of E2F8 was observed in PTC tissues compared with matched adjacent normal tissues (staining score: 202.8 ± 6.182 vs 96.90 ± 6.940; *p* < 0.001) (Fig. 1e and f). Consistent with mRNA results as mentioned above, the score of E2F8 staining was also significantly increased along with more advanced T stage in PTC tissues (Fig. 1e and g).

### Knockdown of E2F8 inhibits PTC cells proliferation and induces cell cycle arrest in vitro

**Fig. 2:**
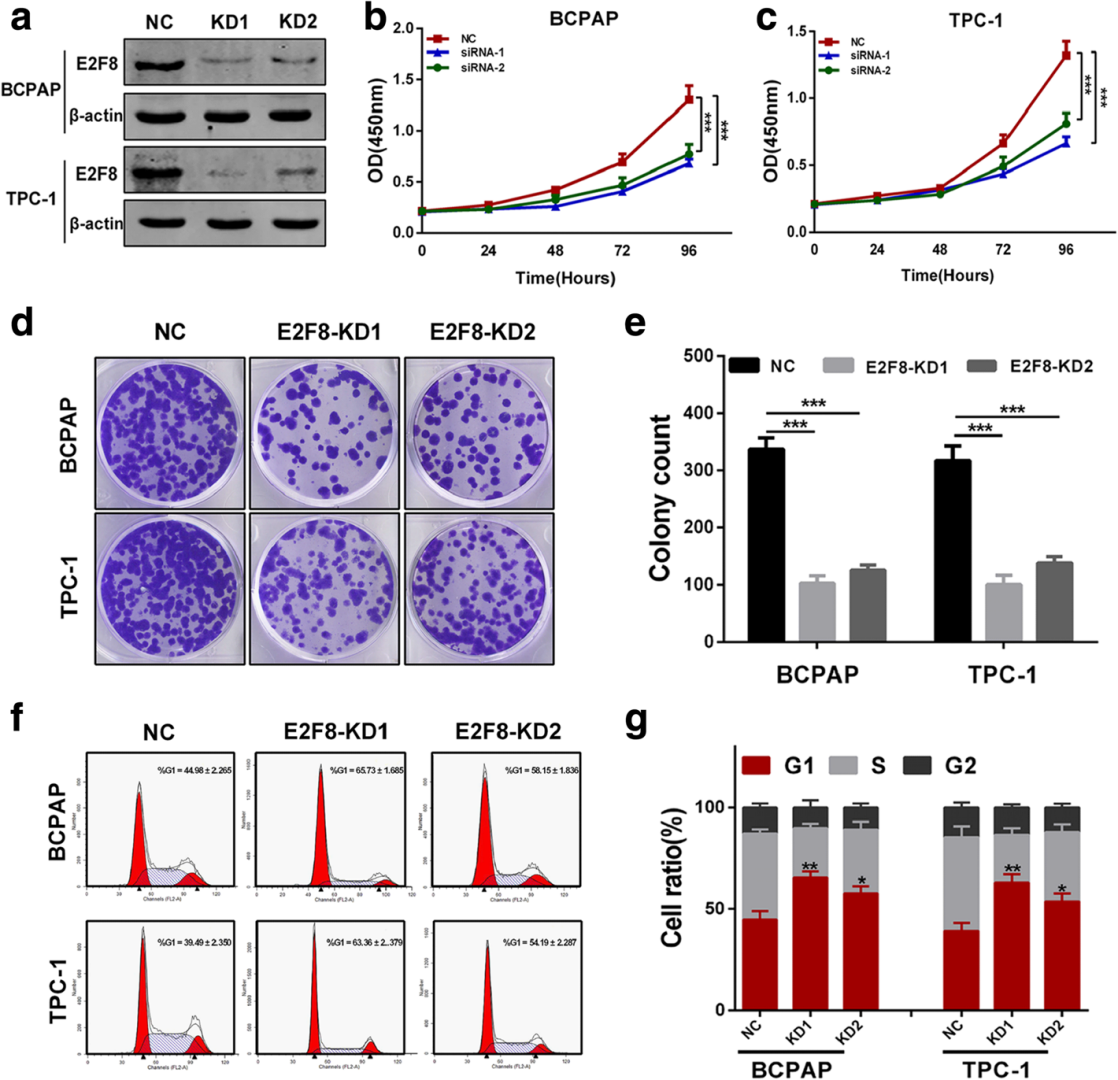
Knockdown of E2F8 inhibits PTC cells proliferation and induces G1-phase arrest in vitro*.* **a** Two different effective siRNAs were used to knockdown E2F8, and the transfection efficiency was measured by western blot in both BCPAP and TPC-1. **b** and **c** Knockdown of E2F8 inhibited both BCPAP and TPC-1 cells proliferation. **d** and **e** Colony numbers of BCPAP and TPC-1 cells transfected with two siRNAs targeting E2F8 were significantly less than those transfected with siRNA-NC. **f** and **g** BCPAP and TPC-1 cells transfected with siRNA-E2F8 exhibited more arrest at G1 phase than those transfected with siRNA-NC. *p < 0.05, **p < 0.01, ***p < 0.001

To investigate the biological function of E2F8 in vitro, two different effective siRNAs were used to knockdown(KD) E2F8, and the transfection efficiency was measured by western blot (Fig. 2a). As shown in Fig. 2b and c, cell-counting kit-8 (CCK-8) assay revealed that knockdown of E2F8 markedly inhibited proliferation of both BCPAP and TPC-1 cells. Moreover, the siRNA-E2F8 transfected groups had significantly fewer colonies than the matched siRNA-NC group (Fig. 2d and e).

Finally, the effect of E2F8 on cell cycle distribution was evaluated by flow-cytometry analysis. As shown in Fig. 2f and g, the percentage of BCPAP and TPC-1 cells transfected with siRNA-E2F8 was significantly increased in G1 phase compared to siRNA-NC.

### Silence of E2F8 suppresses tumor growth in vivo

**Fig. 3:**
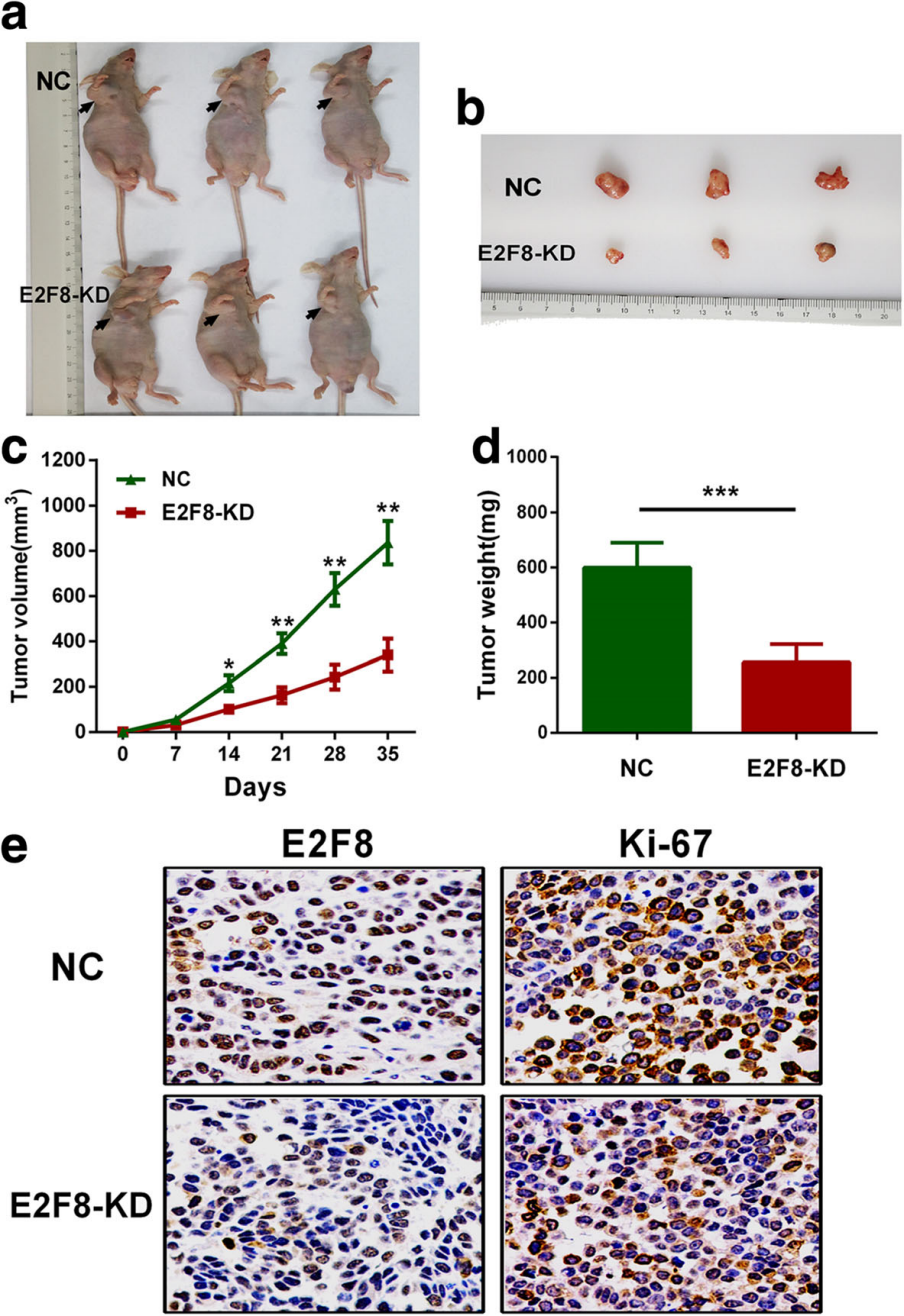
Knockdown of E2F8 inhibits tumor growth in vivo*.* **a** Xenograft model in nude mice. **b** Nodules harvested from shRNA-NC group and E2F8-KD group. **c** and **d** Tumor nodules derived from shRNA-E2F8-transfected TPC-1 cells are significantly smaller than those in shRNA-NC group. **e** IHC analysis of xenograft tumors showed that Ki-67 staining was weaker in shRNA-E2F8 group. *p < 0.05, **p < 0.01, ***p < 0.001

To assess the oncogenic role of E2F8 in vivo, we established xenograft tumor models using TPC-1 cells transfected with shRNA-NC and shRNA-E2F8. First, we observed that all the nude mice developed xenograft tumors at the injection sites, and xenograft tumors were harvested 5 weeks after injection (Fig. 3a and b). Second, as shown in Fig. 3c and d, average volume and weight of tumors in the shRNA-E2F8 group were significantly lower than those in the shRNA-NC group. Finally, IHC analysis revealed that tumors derived from shRNA-E2F8 transfected cells showed weaker staining of Ki-67 than those in the shRNA-NC group (Fig. 3e). These data suggested that increased E2F8 might promote tumor growth in vivo.

### E2F8 exerts its oncogenic activity via influencing CCND1

**Fig. 4:**
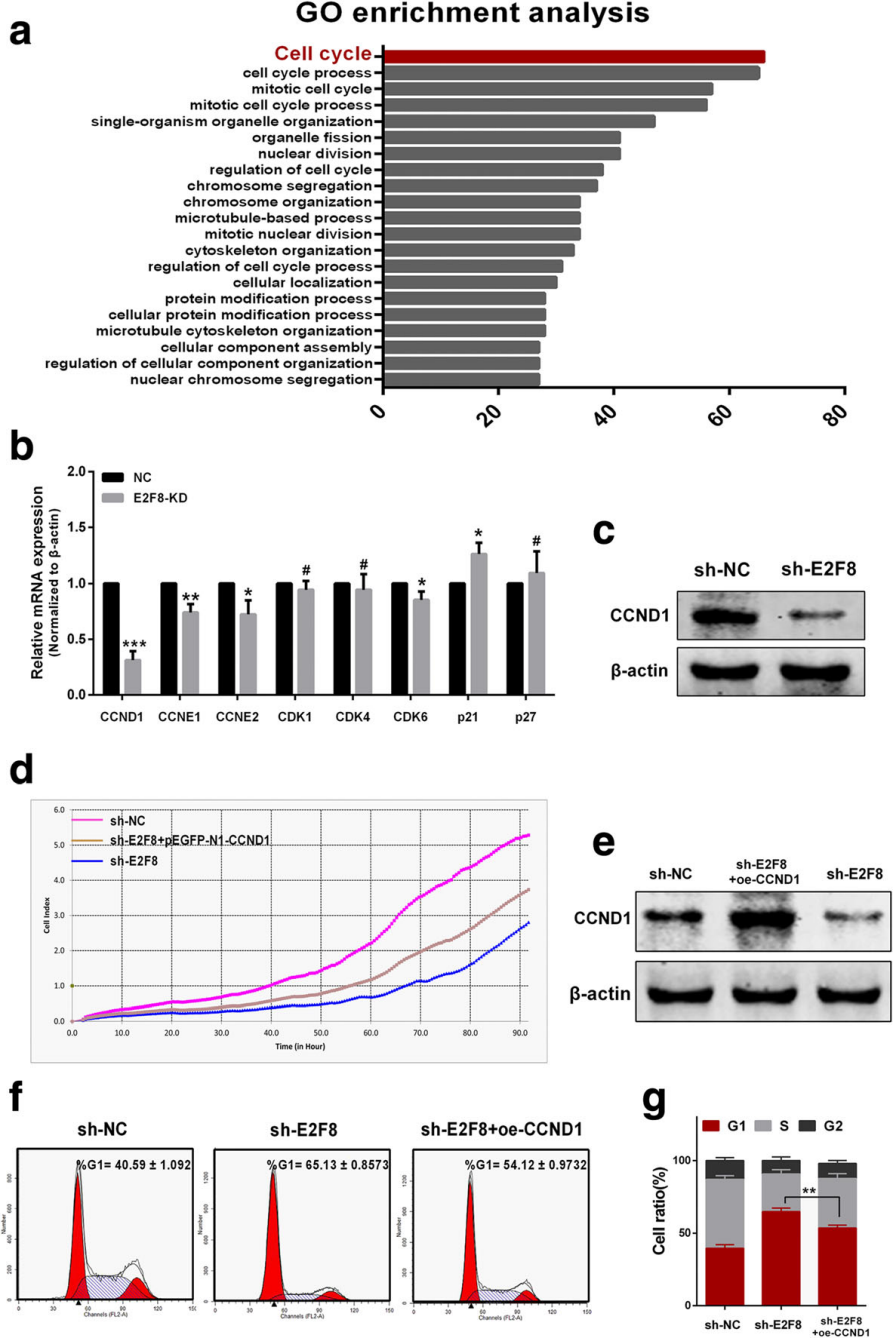
E2F8 promotes cell cycle progression via influencing CCND1 mostly. **a** GO enrichment analysis showed that genes co-expressed with E2F8 are enriched in the “cell cycle” pathway. **b** qRT-PCR analysis of the effect of E2F8-KD on mRNA levels of CCND1, CCNE1, CCNE2, CDK1, CDK4, CDK6, p21 and p27 relative to negative control. **c** Western blot showed that CCND1 protein expression was downregulated in shRNA-E2F8 transfected TPC-1 cells compared with negative control. **d** Enforced overexpression of CCND1 (oe-CCND1) could partially reverse the shRNA-E2F8-mediated proliferation inhibition of TPC-1 cells. **e** Transfection efficiency of CCND1 was determined by western blot. **f** and **g** Flow-cytometry analysis showed that enforced oe-CCND1 significantly alleviated shRNA-E2F8-mediated G1-phase arrest in TPC-1 cells. *p < 0.05, **p < 0.01, ***p < 0.001, # No significance

To explore how E2F8 exerts its oncogenic activity, a list of 143 genes that have highest correlation values with E2F8 were selected from TCGA PTC dataset. By GO enrichment analysis on the 143 genes, as shown in Fig. 4a, the data revealed that most of the genes were enriched in the “cell cycle” pathway. The result indicated that E2F8 might play a pivotal role in the cell cycle.

Considering that knockdown of E2F8 induces G1 phase arrest in vitro, we sought to determine the mRNA levels of CCND1, CCNE1, CCNE2, CDK1, CDK4, CDK6, p21 and p27 which were closely related to G1 phase or G1/S transition in both shRNA-E2F8 or shRNA-NC cells by qRT-PCT. Interestingly, compared with shRNA-NC transfected cells, the result showed that mRNA expression levels of CCND1, CCNE1, CCNE2 and CDK6 were significantly decreased, p21 was significantly increased, while CDK1, CDK4 and p27 were not influenced in shRNA-E2F8 transfected cells (Fig. 4b). Among these genes, considering CCND1 was a key factor to control G1 phase and it was also the most change in mRNA level, we validated its protein level in shRNA-E2F8 cells. By western blot, the data showed that CCND1 expression was markedly decreased in shRNA-E2F8 group compared to shRNA-NC group (Fig. 4c). To examine whether E2F8 regulated the proliferation of TPC-1 cells via altering CCND1, TPC-1 cells with shRNA-E2F8 was transfected with pEGFP-N1-CCND1 plasmid. Transfection efficiency was determined by western blot (Fig. 4e). By the xCELLigence system analysis, the data showed that the proliferation ability was partially recovered in TPC-1 cells with shRNA-E2F8 after enforced overexpression of CCND1(oe-CCND1) (Fig. 4d). Moreover, flow-cytometry analysis showed that oe-CCND1 significantly alleviated shRNA-E2F8-mediated G1-phase arrest of TPC-1 cells (Fig. 4f and g). Our results demonstrated that E2F8 might promote PTC cells proliferation by regulating cell cycle, especially by influencing CCND1.

### E2F8 is specifically targeted by miR-144

**Fig. 5:**
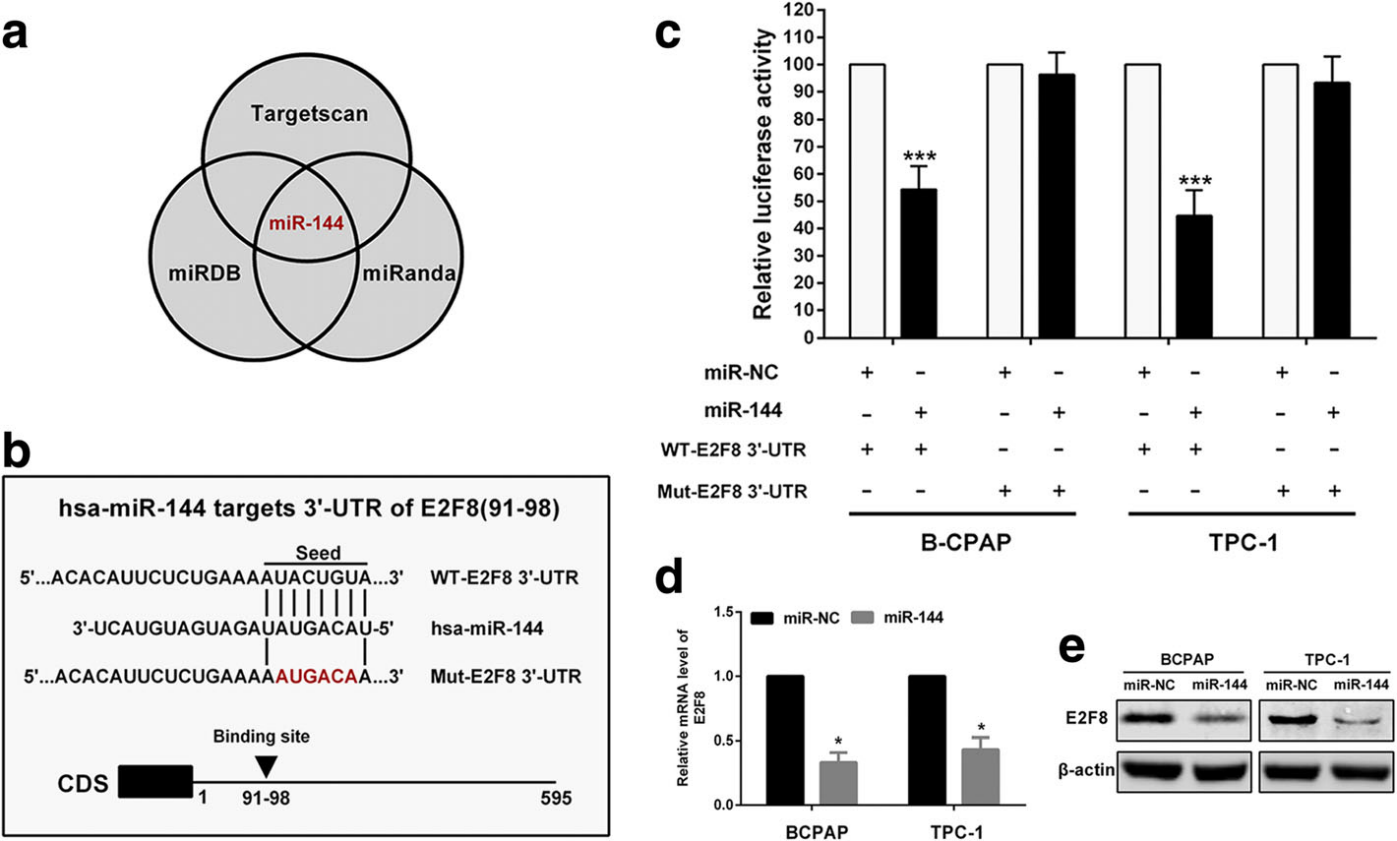
E2F8 is a direct target of miR-144. **a** Venn diagram showing miR-144 targets E2F8 by three prediction software packages. **b** Predicted miR-144 target sequences in E2F8 3’-UTR. Six nucleotides (*red*) were mutated to prevent binding to miR-144. **c** Relative luciferase activity of reporter plasmids carrying wild-type or mutant E2F8 3’-UTR in BCPAP and TPC-1 cells co-transfected with miR-NC or miR-144 mimic. Data were presented as mean ± SD. **d** qRT-PCR. Level of E2F8 mRNA after miR-NC or miR-144 transfected into PTC cells. **e** Western blot. Level of E2F8 protein after miR-NC or miR-144 transfected into PTC cells. *p < 0.05, ***p < 0.001

MiRNAs have been found to play diverse, key biological roles in cancer development and have been widely used for cancer diagnosis, prognosis, and as therapeutic targets. To identify potential miRNAs targeting E2F8, we used a combination of three algorithms, Targetscan, miRDB and miRanda. Among candidate miRNAs, miR-144 was identified by all the three programs as targeting E2F8 with highest predictive score (Fig. 5a). As shown in Fig. 5b, 91–98 of E2F8 3’-UTR was a predicted target of miR-144. Next, we performed luciferase reporter assays to determine whether miR-144 regulates E2F8 expression through binding to the predicted site in 3’-UTR of E2F8 mRNA. As expected, data demonstrated that miR-144 inhibited luciferase activity by around 46% in BCPAP cells and 55% in TPC-1 cells when the reporter plasmid carried the wild-type(WT)-E2F8 3’-UTR, but no significant inhibition was observed at the reporter plasmid carried a mutant(Mut)-E2F8 3’-UTR (Fig. 5c). We then examined the effect of miR-144 on the mRNA and protein expression of E2F8 in BCPAP and TPC-1 cells, respectively. As shown in Fig. 5d and e, results of qRT-PCR and western blot demonstrated that miR-144 inhibited expression of E2F8 compared with negative control group. Our results reveal that miR-144 targets E2F8 by directly binding to the predicted site in 3’-UTR of E2F8 mRNA.

### MiR-144 inhibits proliferation and induces G1 phase arrest in PTC cells

**Fig. 6:**
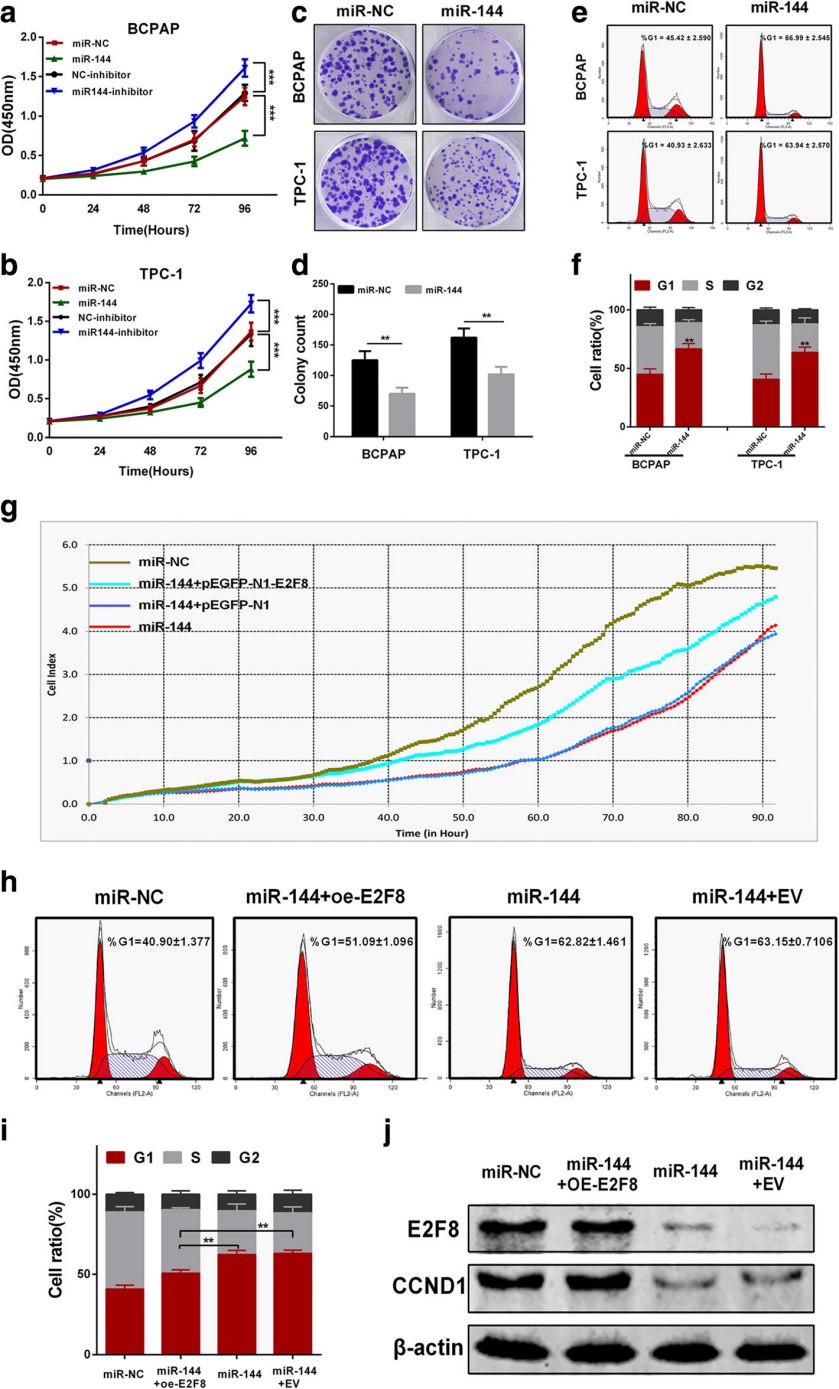
miR-144 inhibits PTC cells proliferation via inducing G1-phase arrest. **a** and **b** Transfection of miR-144 mimic inhibited cells proliferation, while transfection of miR-144 inhibitor promoted cells proliferation in BCPAP and TPC-1 cells. **c** and **d** Colony formation ability was also decreased in both BCPAP and TPC-1 cells after they were transfected with miR-144 compared with miR-NC. **e** and **f** miR-144 significantly caused G1-phase arrest in both BCPAP and TPC-1 cells compared with miR-NC. **g** Effect of enforced overexpression of E2F8 could partially reverse the miR-144-mediated inhibition of proliferation in TPC-1 cells. **h** and **i** Flow-cytometry analysis showed that enforced oe-E2F8 treatment could significantly alleviate miR-144-mediated G1-phase arrest of TPC-1 cells. **j** E2F8 and CCND1 protein levels were determined by western blot in each group. *p < 0.05, **p < 0.01, ***p < 0.001

Finally, we investigated the biological role of miR-144 in vitro. CCK-8 assay showed that the proliferation ability of BCPAP and TPC-1 cells transfected with miR-144 was markedly decreased compared with cells transfected with miR-NC (Fig. 6a and b). Moreover, the colony number of cells with miR-144 was significantly reduced compared to negative control (Fig. 6c and d). Furthermore, the effect of miR-144 on cell cycle distribution was evaluated by flow-cytometry analysis. As shown in Fig. 6e and f, miR-144 transfection treatment significantly caused more BCPAP and TPC-1 cells arrested in G1 phase compared with negative control group.

To test whether restoration of E2F8 could reverse miR-144-mediated inhibition of proliferation of PTC cells, we performed the rescue experiments in TPC-1 cells. We found that enforced overexpression of E2F8(oe-E2F8) by transfection of a cDNA that lacked the miR-144-binding site in the 3’-UTR partially abrogated miR-144-mediated suppression of proliferation in TPC-1 cells (Fig. 6g). As shown in Fig. 6h and i, flow-cytometry analysis also showed that oe-E2F8 partially alleviated miR-144-mediated G1-phase arrest. Expression of E2F8 and CCND1 was verified by western blot in each group (Fig. 6j).

### MiR-144 inhibits tumor growth in vivo

**Fig. 7:**
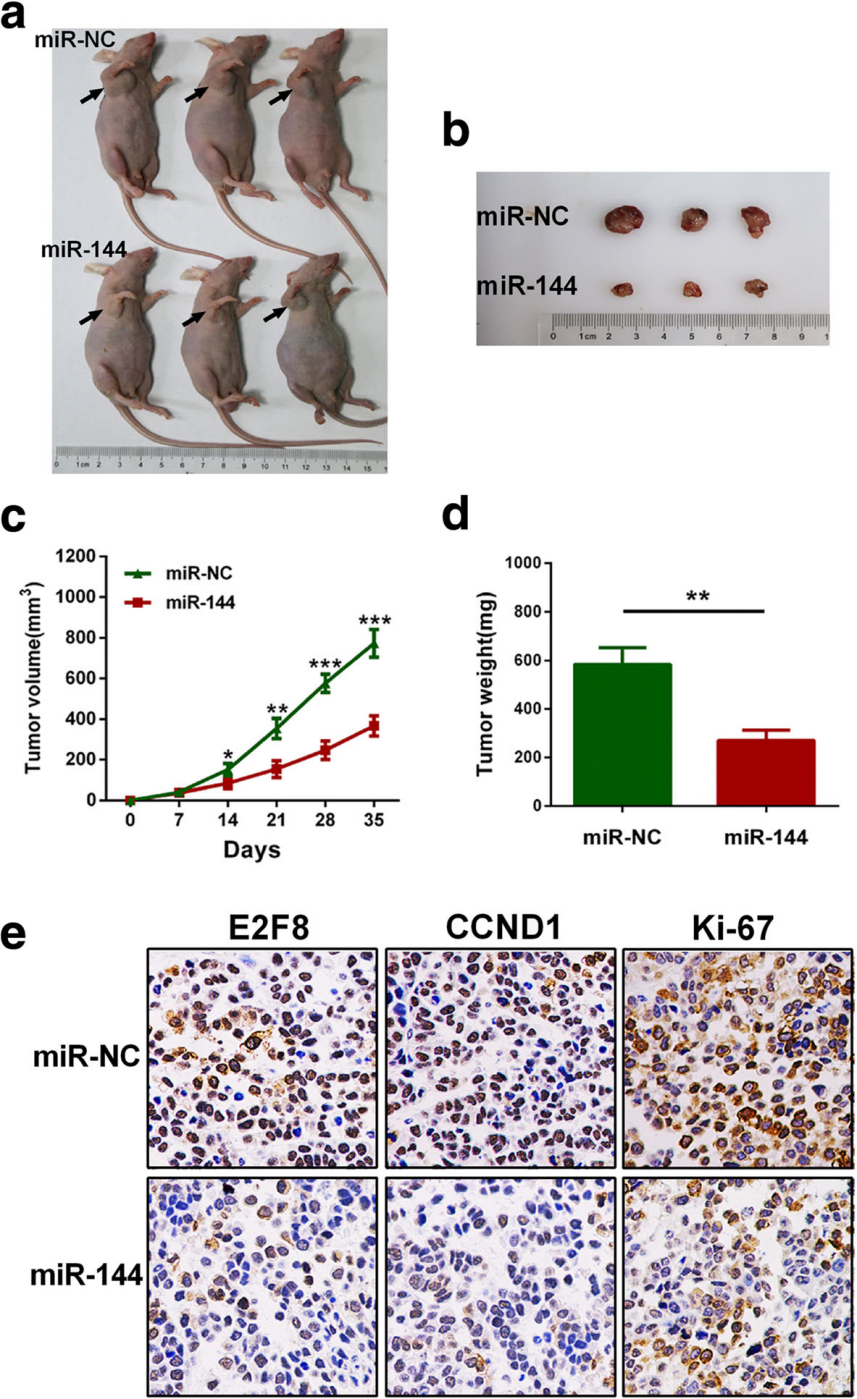
miR-144 inhibits tumor growth in vivo. **a** Xenograft model in nude mice. **b** Tumors harvested from miR-NC group and miR-144 group. **c** and **d** Tumor volumes and weights were significantly reduced in miR-144 group compared with miR-NC group. **e** IHC analysis showed that E2F8 and CCND1 protein levels were both greatly decreased in miR-144 transfected tumors. *p < 0.05, **p < 0.01, ***p < 0.001

To confirm the growth-inhibitory effect of miR-144 on PTC cells in vivo, the subcutaneous growth of tumors derived from TPC-1 cells transfected with miR-144 or miR-NC was assessed. All the nude mice developed xenograft tumors at the injection sites, and xenograft tumors were harvested 5 weeks after injection (Fig. 7a and b). In all mice, the proliferation of tumor cells stably overexpressing miR-144 was dramatically suppressed compared to that with miR-NC. Tumor volumes and weights were significantly reduced in miR-144 transfected tumors compared with NC (Fig. 7c and d). IHC analysis showed that levels of E2F8 and CCND1 protein were greatly decreased in miR-144 transfected tumors (Fig. 7e).

### MiR-144 is downregulated in PTC tissues and inversely correlates with E2F8 expression

**Fig. 8:**
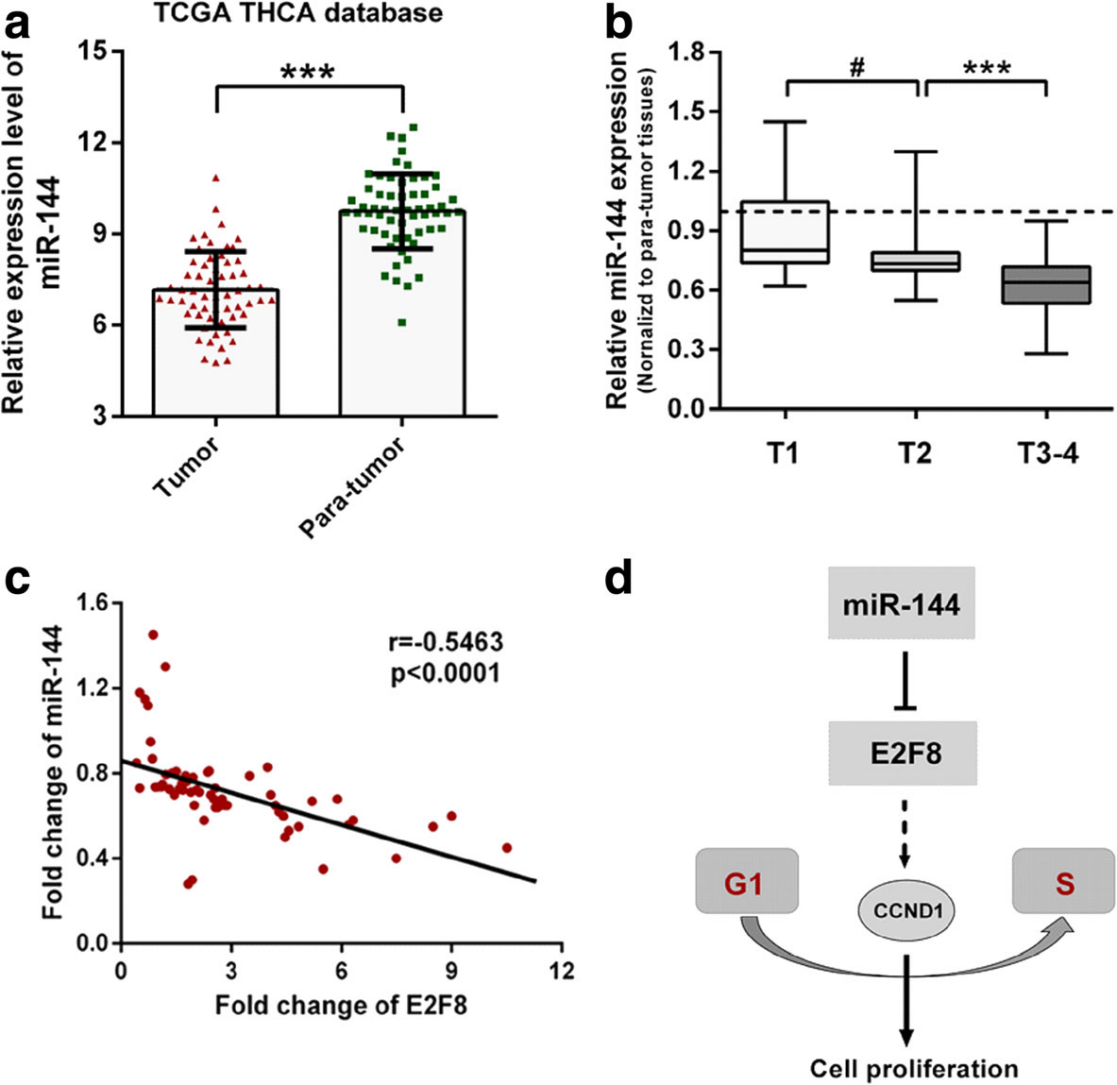
miR-144 is downregulated in PTC tissues and inversely correlates with E2F8 expression. **a** TCGA dataset showed that miR-144 was significantly downregulated in PTC tissues compared with adjacent normal tissues. **b** qRT-PCR analysis showed that miR-144 was significantly decreased along with more advanced T stage in PTC tissues. U6 was used as an internal control to normalize expression data. **c** Pearson correlation scatter plot of fold change in miR-144 levels and E2F8 mRNA levels in PTC tissues (*n* = 64). **d** A hypothetical model of the miR-144/E2F8/CCND1 axis controlling PTC cell proliferation. ***p < 0.001, # No significance

To study the expression pattern of miR-144 in PTC, we first referred to the TCGA dataset named *TCGA_THCA_miRNA_HiSeq-2015-02-24*. In TCGA dataset, miR-144 was found to be significantly downregulated in PTC tissues compared with paired adjacent normal tissues (Fig. 8a). Then, 64 collected paired tissues were used to investigate the association between miR-144 expression and T stage. As shown in Fig. 8b, miR-144 expression was found to be significantly decreased along with more advanced T stage in PTC tissues. Moreover, levels of miR-144 and levels of E2F8 mRNA in PTC tissues exhibited a significant inverse correlation calculated by Pearson correlation test (Fig. 8c).

## Discussion

Sustaining proliferation is thought to be a fundamental hallmark of cancer [27]. Generally, cell cycle progression is dysregulated in cancer cells, thereby breaking homeostasis of cell number and causing uncontrolled cell proliferation [28]. During cell cycle progression, G1/S transition is thought to be a critical step, in which certain cyclins, cyclin-dependent kinases (CDKs) and cyclin dependent kinase inhibitors participate. For instance, CCND1 binds to CDK4 or CDK6, which in turn phosphorylates Rb to promote G1/S transition [29].

E2F proteins have been proved to be important regulators in malignant progression in various cancers. Newly identified E2F8 was reported to be a critical proliferation promoter in several human cancers [14–16]. However, the clinical significance and biological function of E2F8 in papillary thyroid cancer remain unknown.

In this study, we present the first evidence that upregulation of E2F8 occurs widely in PTC, and positively correlates with more aggressive clinicopathological features at both mRNA and protein levels. GO enrichment analysis showed that the term “cell cycle” ranks first among E2F8-related potential pathways. Consistent with this finding, experiments in vitro showed that suppression of E2F8 significantly inhibited cell proliferation via G1-phase arrest. We therefore measured several critical G1-phase genes or G1/S transition regulators to explore the potential mechanism. We found that CCND1 was the most decreased one in shRNA-E2F8 cells. Then rescue experiment was performed, and we found that enforced overexpression of CCND1 could greatly increase proliferation ability and alleviate G1-phase arrest in shRNA-E2F8 cells. CCND1 was a well-documented important regulator that promotes G1/S transition and functions as an oncogene involved in many cancers, including PTC [30–32]. We concluded that E2F8 might exert its proliferative role by influencing CCND1 expression to a great degree in PTC progression.

The development of PTC is regarded as a progressive event involving complicated networks of aberrant gene expression and environmental alteration, in which miRNAs play important roles [33]. In a bioinformatics search for potential miRNAs targeting E2F8, we identified miR-144 as the most promising one. Then, miR-144 was proved to be a specific miRNA targeting E2F8 which has not been previously identified. We found that miR-144 inhibited PTC cells proliferation by decreasing E2F8 posttranscriptionally. The levels of E2F8 mRNA exhibited an inverse correlation with the levels of miRNA-144 in 64 PTC tissues. MiR-144 downregulated the expression of E2F8, and resulted in G1-phase arrest in PTC cells. This result was strongly supported by the rescue experiments in which enforced overexpression of E2F8 could partially reverse cell proliferation inhibition and alleviate G1-phase arrest by miR-144. Our data indicated that miR-144 suppressed PTC cell proliferation at least in part through decreasing the posttranscriptional level of E2F8 (Fig. 8d).

## Conclusions

In this study, we provide evidence that E2F8 functions as a proliferation-related oncogene in PTC progression. Moreover, miR-144 appears to be a tumor suppressor through direct inhibition of E2F8. Our results suggest that the miR-144/E2F8/CCND1 axis might represent a potential therapeutic strategy for treatment of human PTC.

## Additional files

Additional file 1: Table S1. A list of 143 genes with highest co-expression correlation (Pearson *r* value > 0.5) with E2F8 in TCGA PTC dataset. (XLSX 13 kb)
Additional file 2: Table S2. Sequences of qRT-PCR primers. (XLSX 9 kb)

## Abbreviations

CCND1: Cyclin D1
IHC: Immunohistochemistry
PTC: Papillary thyroid cancer
qRT-PCR: Quantitative real-time reverse transcription polymerase chain reaction
TCGA: The Cancer Genome Atlas
TMA: Tissue microarray
WB: Western blot
